# Luteolin Ameliorates Hypertensive Vascular Remodeling through Inhibiting the Proliferation and Migration of Vascular Smooth Muscle Cells

**DOI:** 10.1155/2015/364876

**Published:** 2015-09-30

**Authors:** Jie Su, Han-Ting Xu, Jing-Jing Yu, Jian-Li Gao, Jing Lei, Qiao-Shan Yin, Bo Li, Min-Xia Pang, Min-Xia Su, Wen-Jia Mi, Su-Hong Chen, Gui-Yuan Lv

**Affiliations:** ^1^Zhejiang Chinese Medical University, Hangzhou, Zhejiang 310053, China; ^2^Wenzhou Medical University, Wenzhou, Zhejiang 325035, China; ^3^Zhejiang University of Technology, Hangzhou, Zhejiang 310014, China

## Abstract

*Objectives*. Preliminary researches showed that luteolin was used to treat hypertension. However, it is still unclear whether luteolin has effect on the hypertensive complication such as vascular remodeling. The present study was designed to investigate the effect of luteolin on the hypertensive vascular remodeling and its molecular mechanism. *Method and Results*. We evaluated the effect of luteolin on aorta thickening of hypertension in spontaneous hypertensive rats (SHRs) and found that luteolin could significantly decrease the blood pressure and media thickness of aorta *in vivo*. Luteolin could inhibit angiotensin II- (Ang II-) induced proliferation and migration of vascular smooth muscle cells (VSMCs). Dichlorofluorescein diacetate (DCFH-DA) staining result showed that luteolin reduced Ang II-stimulated ROS production in VSMCs. Furthermore, western blot and gelatin zymography results showed that luteolin treatment leaded to a decrease in ERK1/2, p-ERK1/2, p-p38, MMP2, and proliferating cell nuclear antigen (PCNA) protein level. *Conclusion*. These data support that luteolin can ameliorate hypertensive vascular remodeling by inhibiting the proliferation and migration of Ang II-induced VSMCs. Its mechanism is mediated by the regulation of MAPK signaling pathway and the production of ROS.

## 1. Introduction

Cardiovascular disease is generally regarded as the biggest cause of mortality in the world, and hypertension is mainly associated with increased risk of cardiovascular disease such as coronary artery disease, stroke, and heart failure [1]. An estimated 20% of Chinese adults (>18 years of age) population are in hypertension, exposing these individuals to an increased risk of mortality and cardiovascular events over their lifespan. Under the hypertensive condition, structural remodeling of blood vessels named vascular remodeling has closely participated in the development and maintenance of hypertension and its complications [2–4], which became one of the most serious hypertensive complications.

Vascular remodeling is structural changes of the arterial walls, such as increased intima-media thickness, arterial stiffening, and deteriorating endothelial function [5]. A reduction of lumen ratio and an increase of media-to-lumen ratio are found in almost all hypertensive subjects, as a result of abnormal proliferation, rearrangement of smooth muscle cells, and increased expressions of collagen and fibronectin [6]. Increased arterial wall to lumen diameter ratio may contribute to both enhanced vascular reactivity and vascular stiffness, two cardinal features of hypertension-associated vascular pathology [3, 7]. Furthermore, VSMCs are dynamic, multifunctional cells that act in arterial remodeling through numerous processes, such as cell growth (hyperplasia and hypertrophy), cell migration to the intima, cell apoptosis, reorganization of cells, and altered extracellular matrix composition [8, 9].

Ang II, one of the most important factors in the rennin angiotensin aldosterone system (RAAS), regulates blood pressure and the volume of circulating blood. Dysregulation of Ang II is an important factor contributing to the pathogenesis of hypertension [10]. It has been regarded as a vasoconstrictor agent, which can directly elicit enhanced vasoconstrictor responses in essential hypertension [11]. More importantly, Ang II binds to angiotensin type 1 receptor (AT_1_R) at the cell surface, which induces intracellular generation of reactive oxygen species (ROS) influencing redox-sensitive signaling molecules, such as p38 MAP kinase, ERK1/2, and matrix metalloproteinases (MMPs). The activation of pathways leads to the excessive proliferation and migration of VSMCs, which can cause vascular remodeling [6, 12].

Luteolin (3,4,5,7-tetrahydroxyflavone), a flavonoid, is an important plant compound postulated to be responsible for the biochemical benefits of eating vegetables and fruits and has been reported to possess a variety of biological and pharmacological activities, including antioxidant, anti-inflammatory, anticancer, antiplatelet, and other activities [13–15]. One of the most well-known benefits of luteolin is improving cardiovascular health [16].

Many studies have indicated that luteolin exerted an effect on cardiovascular protection, particularly on hypertension and its related diseases. Accumulating evidences suggested that the blood pressure of rat was directly decreased after oral administration of luteolin [17, 18]. In addition, some researchers found that treatment with luteolin markedly inhibited the impairment of phenylephrine-induced endothelium dependent contraction in aortic rings, which showed that luteolin may be a vascular protective agent [19–21]. Furthermore, a great number of recent advances in cellular biology had demonstrated that luteolin could suppress the proliferation and migration of VSMCs [22]. Endothelial dysfunction is also associated with the pathogenesis of vascular diseases [23, 24], which could be ameliorated by luteolin [25–27]. Hence, these studies showed that luteolin could exert effect on hypertensive vascular remodeling by protection of blood vessel.

In our preliminary experiment, we found that oral administration of luteolin at 25 mg/kg with buddleoside could significantly inhibit the blood pressure on SHR [28]. We further clarified that its effective dosage for antihypertensive treatment is about 75 mg/kg. Nevertheless, it was still uncertain whether luteolin was also effective in hypertensive complication such as vascular remodeling. Therefore, in this study, we employed SHR model to evaluate whether luteolin can inhibit the hypertensive vascular remodeling* in vivo*. Furthermore, we sought to clarify its molecular mechanism of action using Ang II-induced VSMCs model* in vitro*. The purpose of this study was to elucidate the mechanism of luteolin against cardiovascular complications caused by hypertension.

## 2. Materials and Methods

### 2.1. Cell Culture and Animals

VSMCs were isolated from thoracic aortas of Sprague-Dawley rats, which were purchased from Animal Supply Center of Zhejiang Academy of Medical Science (certificate number SCXK2008-0033, Hangzhou, China) following the tissue explants method as previously described [29]. VSMCs were allowed to grow from the explants for 7–10 days and grew in a typical “hill-and-valley” pattern. Cells were maintained in RPMI 1640 Medium supplemented with 20% FBS (Invitrogen, Carlsbad, CA, USA), 1 mM sodium pyruvate, 100 U/mL penicillin, and 100 *μ*g/mL streptomycin at 37°C in a 5% CO_2_ incubator. Early subcultured cells (from passage 2–5) were used in the experiments.

Male SHRs, age of 12 weeks, and Wistar-Kyoto (Wky) rats, age of 12 weeks, were obtained from Vital River Laboratories (certificate number SCXK2012-0001, Beijing, China) and acclimatized for at least two weeks. During this period, the rats were supplied with tap water and rodent laboratory chow ad libitum, as well as a daily health inspection under a controlled room with stable temperature, humidity, and light/dark cycle. All the procedures were in strict accordance following the guidelines for the Use and Care of Laboratory Animals published by the Zhejiang province (2009).

### 2.2. Drug and Chemicals Preparation

Luteolin (LUT, purity > 98%) was purchased from Shanghai Tauto Biotech Co., Ltd. (Shanghai, China). Valsartan (Val, purity > 98%) was purchased from Sigma-Aldrich (St. Louis, MO, USA). These compounds for experiments* in vitro* were dissolved in dimethyl sulfoxide (DMSO) to make stock solutions and were kept at −20°C as aliquots. The stock solution was diluted with serum-free medium before use. Valsartan Capsules, which from Beijing Novartis Pharma Co., Ltd. (Beijing, China, CHN Lot: x1542), were dissolved with distilled water into 0.8 mg/mL for positive control.

**Figure 1:**
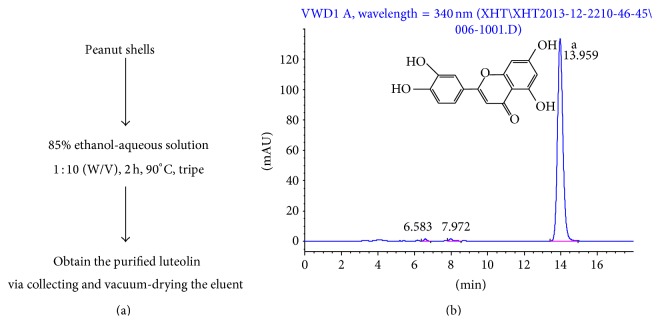
(a) The route diagram of preparing luteolin enriched extracts. (b) HPLC chromatogram of luteolin enriched extracts detected at 340 nm. Peak a was identified to be luteolin.

Luteolin enriched extracts (LUT50, purity > 50%) were extracted from peanut shells purchased from New Nongdu Co., Ltd. (Hangzhou, China) (see Figure 1(a)).

### 2.3. HPLC-DAD Analysis

All the crude extracts of peanut shells were analyzed with HPLC-DAD. The analysis was similar as described by Lv et al. [28]. Simply speaking, sample concentrations were 6.20 mg of extract in 25 mL of methanol and filtered through a 0.45 *μ*m membrane filter before delivered into the system. The Agilent HPLC1200 (Agilent Technologies Inc., Palo Alto, USA) was used to determinate the content of luteolin in extracts by Kromasil 100-5 C18 (250 mm × 4.6 mm) column. The mobile phase was composed of methanol, water, and acetic acid (50 : 49 : 1, V : V : V). The solvent flow rate was 1 mL/min and the column temperature was set at 25°C. The injection volume was 5 *μ*L. The photodiode array detector was set at 340 nm with a total runtime of 25 min. The HPLC chromatogram of extracts was shown in Figure 1(b).

### 2.4. Cytotoxicity Assay

MTT was used to measure the viability of VSMCs [30]. VSMCs in the logarithmic growth phase were digested and inoculated in 96-well plates. Each well contained 1.4 × 10^4^ VSMCs in suspension. Cells were placed in serum-free media with various concentrations of LUT (0.1, 0.2, 0.3, 1, 2, 3, 5, 10, 20, and 30 *μ*M) for 24 h. MTT solution (5 mg/mL) was added to each well. Following a 4 h incubation at 37°C, the cell culture medium was removed and 150 *μ*L of DMSO was added to each well. The absorbance of each well was measured with a 96-well microplate reader with the detection wavelength set at 570 nm. The viability of cells in the experimental groups was expressed as a percentage of the viability of control cells (which was taken to be 100%).

### 2.5. Cell Proliferation Assay

The effect of luteolin on cell proliferation was estimated with a modified MTT assay as described in the paper. VSMCs (1.4 × 10^4^ cells/well, 50–70% density) were counted and seeded in 96-well plates. Cells were stimulated with 1 *μ*M Ang II (Merck KGaA, Darmstadt, Germany) in the absence or presence of LUT (1, 2, 3, 5, 10, 20, and 30 *μ*M) during 24 h and followed the conventional procedure. The absorbance of 96-well culture plates was measured at 570 nm.

### 2.6. Crystal Violet Viability Assay

Experimentally, VSMCs (8.0 × 10^4^ cells/well, 50–70% density) were seeded in 24-well plates. VSMCs were incubated with or without LUT (5, 10, and 20 *μ*M) in the presence of Ang II (1 *μ*M) for 24 h. At 24 h after treatment, cells were carefully washed with PBS and stained with 0.5% crystal violet formalin solution at room temperature for 20–30 min. The stained cells were washed with tap water and air-dried for taking macrographic images. For quantitative measurement, the stained cells were dissolved in 20% acetic acid at room temperature for 20 min with shaking. Absorbance at 570 nm was measured.

### 2.7. Monolayer-Wounding Cell Migration Assay

To evaluate the impact of luteolin on cell migration ability, a wound-healing model was used [31]. VSMCs (8.0 × 10^4^ cells/well, 50–70% density) were seeded in 24-well plates and grew to be subjected to wounding. Then cell layers were wounded with a sterile 200 *μ*L pipette tip. After washing away suspended cells, different concentrations of LUT (5, 10, and 20 *μ*M) with Ang II (1 *μ*M) were added into wells for 24 h. Images were photographed in each well at 100x magnification before and after 24 h drug treatment and analyzed with Image-Pro Plus 5.1 software. Average scraped width per well before and after 24 h drug treatment was measured. Migration distance was estimated based on the scraped width of well before and after drug treatment.

### 2.8. Boyden Chamber Transwell Migration Assay

The migration of the cultured cells was examined using a transwell chamber with a polycarbonate membrane (8 *μ*m pores) [32]. The VSMCs were suspended in serum-free RPMI 1640 (2.4 × 10^5^ cells/mL). Then a 250 *μ*L cell suspension (containing different concentrations of LUT simulated by Ang II) was added to the upper chamber, with 10% FBS RPMI 1640 Medium (500 *μ*L) placed in the lower chamber in the absence of cells. The transwell plate was incubated at 37°C in 5% CO_2_ for 24 h. The cells migrated through the micropores, and the migrated cells attached to the lower surface of the transwell filter. After 24 h, the inserts were washed with PBS; upper surface cells were removed by cotton swabs and the lower side was fixed in 4% paraformaldehyde. The migrated cells were then stained with propidium iodide (PI). Three visual fields that were randomly selected from each of the transwell filters were captured at 200x magnification with an inverted fluorescence microscope, and the average number of cells that migrated through the transwell filters was counted under Image-Pro Plus 5.1 software.

### 2.9. Cell Cycle Analysis by Flow Cytometry

Cell cycle regulation was determined by flow cytometry [33]. Cells (4.0 × 10^5^/well) were plated into dishes (60 × 15 mm) 1 day before treatment with LUT (5, 10, and 20 *μ*M) in the presence of Ang II. After treatment for 24 h, cells were harvested, washed with PBS, fixed in cold 70% alcohol overnight at −20°C for at least 2 h, and stained with 50 ng/mL PI in the presence of 200 *μ*g/mL RNase A by incubation at 37°C for at least 30 min. The stained cells were analyzed by flow cytometry (Millipore). Data were analyzed using FlowJo 7.6.1 software.

### 2.10. ROS Assay

ROS was detected under the manual's direction of ROS detection kit (Beyotime, Shanghai, China) [34]. Experimentally, cells (1 × 10^5^/well) were seeded in dishes (35 × 10 mm). And VSMCs were pretreated with the indicated concentration of LUT for 24 h. At 24 h after pretreatment, cells were stained with 40 *μ*M DCFH-DA by incubation at 37°C for 20 min. Then Ang II (1 *μ*M) was added to the dishes for 5 min. Finally, pictures were captured at 100x magnification with an inverted fluorescence microscope, and the intensity of fluorescence was analyzed and quantified using Image-Pro Plus 5.1 software.

### 2.11. Animal Treatments

Seven Wky rats and twenty-eight SHRs were randomly assigned to five groups. The first group (group 1, G1) and the second group (group 2, G2) were, respectively, set as Wky control group and SHR control group, both of which were given distilled water by oral administration. Valsartan (8 mg/kg, p.o.) was given to the third group (group 3, G3) for 6 weeks daily. The fourth group (group 4, G4) and the fifth group (group 5, G5) were received LUT50 (at the doses of 75 and 150 mg/kg, p.o., resp.) for 6 weeks. Throughout the experiment, body weight was evaluated.

### 2.12. Blood Pressure Measurement

In these five groups, doses were administered orally using an oral tube once daily for 6 consecutive weeks, and blood pressure was measured after administration at 6th weeks. Using a noninvasive method of tail-cuff plethysmography (Shanghai Alcott Biotech Co., Ltd., Shanghai China), the systolic, diastolic, and mean arterial blood pressures (SBP, DBP, and MAP for short) were measured at 2 h after administration. Each animal was placed in a 28°C warmer for several minutes. For each time point, four continuous blood pressure values were tested and averaged.

### 2.13. Enzyme-Linked Immunosorbent Assay (ELISA) for Ang II

At the end of the treatment, all rats were fasted overnight and the blood samples were collected via the rat ophthalmic venous plexus. All of the blood samples were centrifuged at 3500 rpm for 10 min, and the serum was separated to determine Ang II activity by the method of ELISA. All of the procedures were performed as described in the assay kit (Shanghai Xinfang Biological Pharmaceutical Technology Co., Ltd., Shanghai, China).

### 2.14. Histological Evaluation

The thoracic aortas were resected and placed in 4% neutral buffered formalin. After fixation, tissues were paraffin-embedded and cut into 4 *μ*m sections. Then sections were stained with hematoxylin and eosin (Nanjing Jiangcheng Bioengineering Institute, Nanjing, China) [35]. Images were captured with the microscope (40x). The thickness of the aorta was measured with Image-Pro Plus 5.1 software. The media thickness was determined by measuring the distance from the internal elastic lamina to the external elastic lamina. For each slide, measurements from 4 points (12, 3, 6, and 9 o'clock positions) were averaged. The lumen inner diameter was determined from 2 points (12 and 9 o'clock positions). The media-to-lumen ratio was calculated based on the measured lumen inner diameter and media data.

### 2.15. Gelatin Zymography

The thoracic aorta samples were homogenized in RIPA buffer (Solarbio, Beijing, China). After centrifugation, clear supernatant was collected. Tissue protein was mixed with 5× nonreducing sample buffer and loaded onto 7.5% polyacrylamide gels containing 0.1% gelatin gels (20 *μ*L/sample), and electrophoresis was performed at 100 V for 4 h at 4°C [36]. After electrophoresis, the gel was rinsed with washing buffer for 1.5 h with shaking at room temperature. The buffer was then changed to incubation buffer and incubated for 48 h at 37°C. Gelatin gel was stained with coomassie blue and then destained with 10% acetic acid. The unstained bands correspond to the areas of gelatin digestion.

### 2.16. Cell Protein Extraction and Western Blotting Analysis

Cells were collected and lysed in RAPI buffer (Solarbio, Beijing, China) with protease/phosphatase inhibitor (Cell Signaling Technology, Canada). After treatment on ice for 30 min, lysates were clarified by centrifugation at 12000 rpm for 15 min at 4°C and the protein content was measured using a BCA protein assay kit (Beyotime, Jiangsu, China). Sample protein was mixed with 5× Loading Buffer (Beyotime, Jiangsu, China). The western blot was similar as described by Gao et al. [36]. In brief, the samples were separated by SDS-PAGE and electrotransferred onto a polyvinylidene-difluoride membrane (Pall Corporation, Mexico). The membrane was blocked with BSA blocking buffer for two hours at room temperature, incubated overnight at 4°C with interest primary antibodies (Santa Cruz Biotechnology, USA, or Cell Signaling Technology, Canada) in PBST. After washing, the membrane was incubated with an appropriate secondary antibody (Santa Cruz Biotechnology, USA) for 30 min. The membrane was incubated with streptavidin HRP (Thermo, USA) for 30 min after washing. The blotted protein bands were detected by Chemiluminescent Substrate kit (BIO-RAD, USA).

### 2.17. Statistical Analysis

All values were expressed as mean ± standard deviation and subjected to one-way analysis of variance (ANOVA) by using SPSS 17.0 for windows. The LSD *t*-tests will be applied when homogeneity of variance assumptions is satisfied; otherwise, the Dunnet *t*-test will be used. A value of *P* < 0.05 was considered to be statistically significant.

## 3. Results

### 3.1. Luteolin Inhibits Ang II-Induced VSMC Proliferation

To clarify the effects of luteolin on vascular remodeling* in vitro*, rat aortic smooth muscle cells were explanted and subjected to examination. We examined the cytotoxicity of luteolin and its inhibitory effects on cell viability in VSMCs with stimulation of Ang II using MTT assay and crystal violet staining to assess the antiproliferation effect.

**Figure 2:**
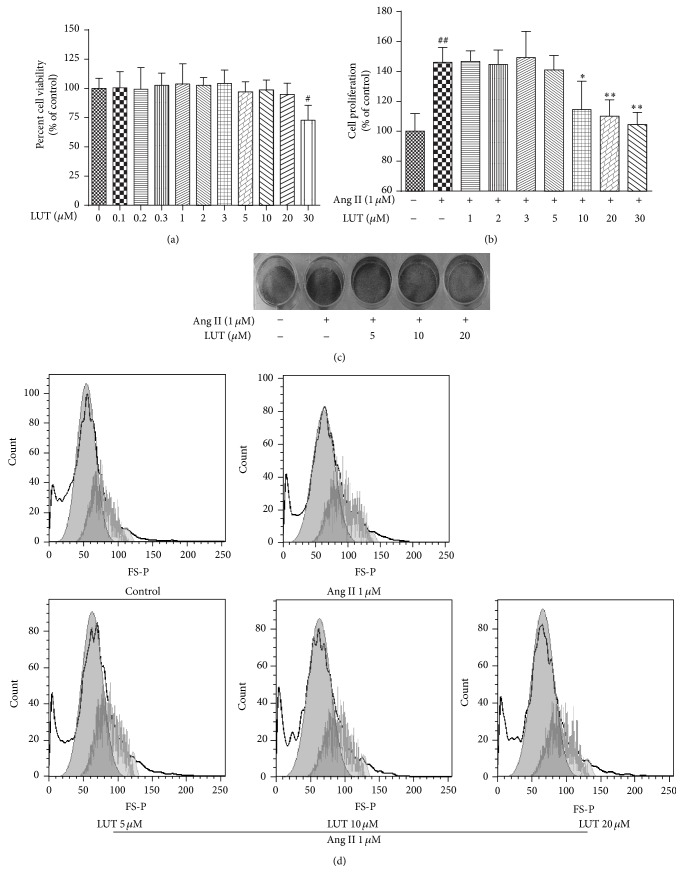
The inhibitory effects of luteolin on the Ang II-induced proliferation of VSMCs. (a) Cytotoxicity of luteolin on VSMCs. The data were expressed as mean ± SD (*n* = 8). (b) Antiproliferative activity of luteolin in VSMCs by MTT assay. The data were expressed as mean ± SD (*n* = 8). (c) Antiproliferative activity of luteolin in VSMCs by crystal violet viability assay. (d) Cell cycle phase analysis. ^#^ *P* < 0.05 versus control group; ^##^ *P* < 0.01 versus control group; ^*∗*^ *P* < 0.05 versus Ang II-treated group; ^*∗∗*^ *P* < 0.01 versus Ang II-treated group.

The cytotoxicity of luteolin was presented in Figure 2(a). The viability of cell administrated of LUT at 30 *μ*M was markedly inhibited in comparison with control (*P* < 0.05). However, other groups' cell viability had no significant difference compared to control. The results suggested that a cytotoxic effect of luteolin was at a concentration of up to 30 *μ*M. In addition, as shown in Figure 2(b), 1 *μ*M Ang II significantly stimulated VSMCs proliferation compared with the control group (*P* < 0.01). However, the action of Ang II was inhibited by LUT at the concentrations of 10 *μ*M (*P* < 0.05), 20 *μ*M, and 30 *μ*M (*P* < 0.01). LUT (<10 *μ*M) did not have any remarkable effect on VSMCs' proliferation. Taking into account the fact that LUT at 30 *μ*M had a cytotoxic effect, LUT at 5 *μ*M, 10 *μ*M, and 20 *μ*M was used in the following experiments. Furthermore, the result of crystal violet staining illustrated in Figure 2(c) proved that Ang II-induced VSMCs proliferation was suppressed by LUT.

To examine the possible mechanisms behind luteolin's inhibition effect on VSMCs' proliferation, we performed cell cycle analysis by FACS. As shown in Figure 2(d)  and Table 1, 1 *μ*M Ang II resulted in an accumulation of cells in the S phase, from 25.63% to 30.22%, and an attenuation of cells in G_2_ phase, from 3.15% to 1.87% compared to control. When VSMCs stimulated by 1 *μ*M Ang II were treated with LUT for 24 h, LUT at 10 *μ*M induced a depletion of cells in the S phase, from 30.22% to 24.72%, and a concomitant accumulation of cells in G_2_ phase, from 1.87% to 2.68%. LUT at 20 *μ*M also induced a depletion of cells in the S phase, from 30.22% to 24.27%, and a concomitant accumulation of cells in G_2_ phase, from 1.87% to 2.79%. LUT at 5 *μ*M had not induced significant change. These data suggested that luteolin could inhibit VSMCs' proliferation.

### 3.2. Luteolin Suppresses Ang II-Induced VSMC Migration

Because VSMCs' migration plays an important role in vascular remodeling and hypertension-associated vascular changes, we determined whether luteolin could suppress Ang II-induced VSMCs migration.

**Figure 3:**
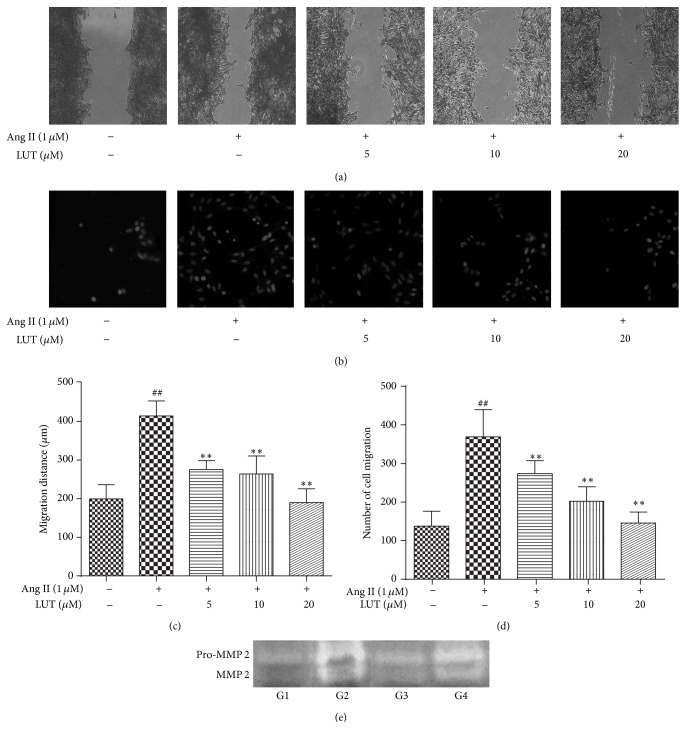
The inhibitory effects of luteolin on the Ang II-induced migration of VSMCs. (a) Monolayer-wounding cell migration assay. Views were photographed along the scraped line in the well at 100x magnification. (b) Boyden chamber transwell migration assay. Images were captured at 200x magnification. (c) The migration distance was tested by monolayer-wounding cell migration assay. The data were expressed as mean ± SD (*n* = 9). (d) The number of cell migration was tested by Boyden chamber transwell migration assay. The data were expressed as mean ± SD (*n* = 9). (e) Gelatin zymography. ^##^ *P* < 0.01 versus control group; ^*∗∗*^ *P* < 0.01 versus Ang II-treated group. G1 = Wky control group, G2 = SHR control group, G3 = valsartan group, and G4 = 75 mg/kg LUT50 group.

As illustrated in Figures 3(a) and 3(c), VSMCs stimulated by 1 *μ*M Ang II were markedly promoted to migrate from one side of the scratch to another side compared with the control group (*P* < 0.01). We discovered that after VSMCs were treated with LUT for 24 h, the Ang II-induced migration of VSMCs was significantly suppressed by 5 *μ*M, 10 *μ*M, and 20 *μ*M LUT (*P* < 0.01). Meanwhile, the result of the Boyden chamber transwell assay illustrated in Figures 3(b) and 3(d) proved that luteolin could inhibit Ang II-induced VSMCs migration. 1 *μ*M Ang II markedly promoted the migration of VSMCs from the upper chamber to the lower chamber in comparison with the control group (*P* < 0.01). When VSMCs were treated with 5 *μ*M, 10 *μ*M, and 20 *μ*M LUT, the numbers of Ang II-induced migrated cells across the extracellular matrix protein-coated membranes were significantly decreased (*P* < 0.01).

MMPs are responsible for matrix degradation, necessary for efficient cell migration during vascular remodeling. To determine the effect of luteolin on the production of MMPs in rat aorta, gelatin zymography was used. As shown in Figure 3(e), pro-MMP 2 (Figure 3(e), top line) and MMP 2 (Figure 3(e), bottom line) enzyme activities in SHR control group were markedly increased in comparison with Wky control group. We found that the expression of pro-MMP 2 and MMP 2 was apparently reduced after SHRs were administrated by valsartan. Meanwhile, treatment with the dose of 75 mg/kg LUT50, the activation of pro-MMP 2 and MMP 2 were significantly suppressed.

### 3.3. Luteolin Inhibits Ang II-Induced Oxidative Stress in VSMCs

ROS are prime candidates in the etiology of vascular remodeling and ensuing cardiovascular disease. To elucidate whether Ang II increases ROS generation and whether luteolin ameliorates this effect, we determined ROS production in VSMCs by DCFH-DA staining.

**Figure 4:**
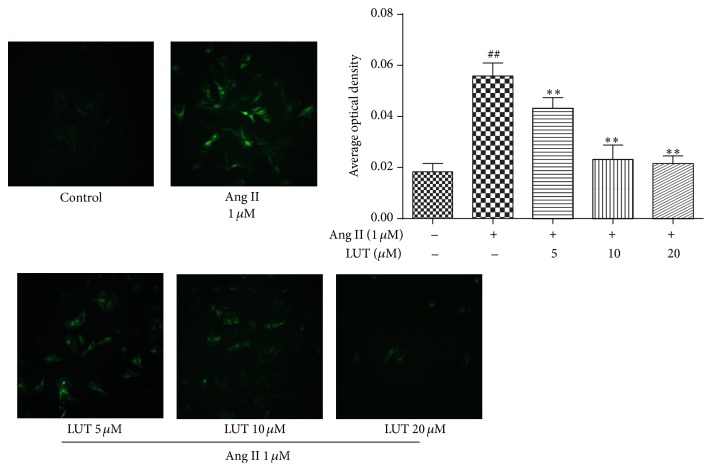
The protective effect of luteolin on Ang II-induced oxidative stress in VSMCs. Images were captured at 100x magnification. The data were expressed as mean ± SD (*n* = 9). ^##^ *P* < 0.01 versus control group; ^*∗∗*^ *P* < 0.01 versus Ang II-treated group.

As presented in Figure 4, compared with control group, the Ang II-stimulated VSMCs exhibited impressively increased DCF fluorescence intensity (*P* < 0.01). However, the effect of Ang II was markedly suppressed in VSMCs treated with 5 *μ*M, 10 *μ*M, and 20 *μ*M LUT (*P* < 0.01). Hence, Ang II induced increase of ROS production, which resulted in oxidative stress. Ultimately, this effect of Ang II could be inhibited by luteolin.

### 3.4. Luteolin Attenuates Hypertension in SHR

To evaluate the direct effect of luteolin on blood pressure of SHR, we examine the blood pressure of SHR after 6 weeks of treatment with LUT50. We had reported that 25 mg/kg LUT50 did not lower the SBP or DBP of the SHRs significantly compared to SHR control group [28]. Hence, in this experiment, the dosage was enlarged to 75 mg/kg and 150 mg/kg.

**Figure 5:**
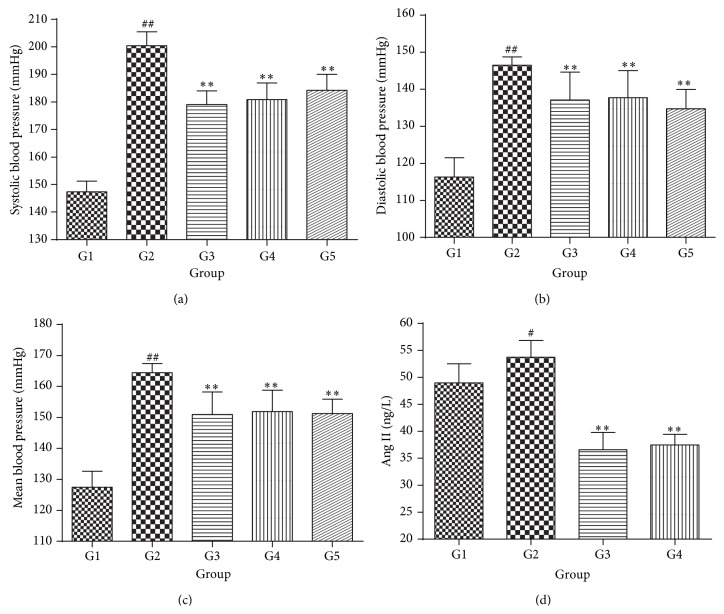
The antihypertensive effect of luteolin extracts in SHRs. (a) The effect on SBP. The data were expressed as mean ± SD (*n* = 7). (b) The effect on DBP. The data were expressed as mean ± SD (*n* = 7). (c) The effect on MBP. The data were expressed as mean ± SD (*n* = 7). (d) The Ang II level in serum. The data were expressed as mean ± SD (*n* = 6). ^#^ *P* < 0.05 versus Wky control group; ^##^ *P* < 0.01 versus Wky control group; ^*∗∗*^ *P* < 0.01 versus SHR control group. G1 = Wky control group, G2 = SHR control group, G3 = valsartan group, G4 = 75 mg/kg LUT50 group, and G5 = 150 mg/kg LUT50 group.

As shown in Figures 5(a), 5(b), and 5(c), the SBP, DBP, and MBP of SHR control group were all obviously increased in comparison with Wky control group (*P* < 0.01). However, after administration of 8 mg/kg valsartan, the SBP, DBP, and MBP of SHR control group were all significantly decreased (*P* < 0.01). Both 75 mg/kg and 150 mg/kg LUT50 also showed a tendency to decrease the SBP, DBP, and MBP of SHRs (*P* < 0.01). Compared to SHR control group, the SBP of 75 mg/kg LUT50 group was decreased 19 mmHg, and the SBP of 150 mg/kg LUT50 group was reduced 16 mmHg. Furthermore, 75 mg/kg LUT50 markedly decreased DBP 8 mmHg of SHR, and 150 mg/kg LUT50 also significantly declined DBP 11 mmHg of SHR. These data suggested that lower dosage of luteolin was good at decreasing the SBP, while that higher dosage of luteolin was adept in declining the DBP. No matter what dose was more effective, it is clear that luteolin treatment attenuated hypertension of SHR.

### 3.5. Luteolin Improves Rat Aorta Vascular Remodeling

The media-to-lumen ratio was used as an index of aortic vascular remodeling. Hence, to evaluate the effect of luteolin on vascular remodeling of SHR, we determined the media thickness of vascular wall and lumen inner diameter in rat aorta using the HE staining.

**Figure 6:**
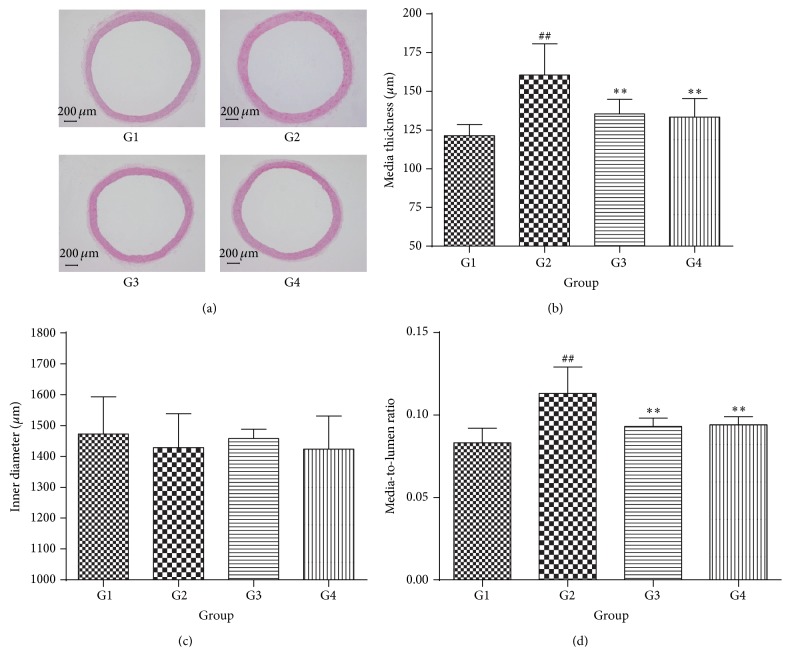
Effect of luteolin on rat aortic remodeling. The data were expressed as mean ± SD (*n* = 6). (a) Representative images of vessel sections stained with HE staining at 40x magnification. (b) The media thickness. (c) The inner diameter. (d) The media-to-lumen ratio. ^##^ *P* < 0.01 versus Wky control group; ^*∗*^ *P* < 0.05 versus SHR control group; ^*∗∗*^ *P* < 0.01 versus SHR control group. G1 = Wky control group, G2 = SHR control group, G3 = valsartan group, and G4 = 75 mg/kg LUT50 group.

The HE staining for rat aorta tissues was presented in Figure 6. As shown in Figure 6(b), the media thickness of vascular wall in SHR was significantly increased compared to Wky control group (*P* < 0.01). The increased media thickness in SHR indicated the progressive worsening of thoracic aorta vascular remodeling by hypertension. However, 8 mg/kg valsartan treatment markedly decreased the media thickness in SHR (*P* < 0.01). Meanwhile, it is pleasant that the media thickness in SHR was apparently decreased after administration of 75 mg/kg LUT50 (*P* < 0.01). Of note, as illustrated in Figure 6(c), there was no significant difference in the lumen inner diameter between SHR and Wky control group with or without treatment with LUT50. Furthermore, as shown in Figure 6(d), there were significant changes in the media-to-lumen ratio of SHR control group in comparison with Wky control group (*P* < 0.01). As excepted, the media-to-lumen ratio was markedly declined in the 75 mg/kg LUT50 or 8 mg/kg valsartan group compared to the SHR control group (*P* < 0.01).

### 3.6. The Potential Drug Targets for Antiremodeling by Luteolin

Ang II, a well-known activator of this signaling pathway, plays a critical role during hypertensive vascular remodeling. In addition, the mitogen-activated protein kinase (MAPK) cascade, particularly the p38 MAP kinase, may play a role in mediating responses that are related to vascular remodeling. To explore the antiremodeling mechanisms of luteolin, we examined the expression of related factors using western blot and ELISA.

Firstly, we detected the Ang II level in serum by ELISA. As shown in Figure 5(d), compared to Wky control group, Ang II level in serum of SHR was apparently increased (*P* < 0.05). Conversely, Ang II level of 8 mg/kg valsartan group was significantly decreased in comparison with SHR control group (*P* < 0.01). As expected, 75 mg/kg LUT50 markedly diminished Ang II level in serum of SHR (*P* < 0.01).

**Figure 7:**
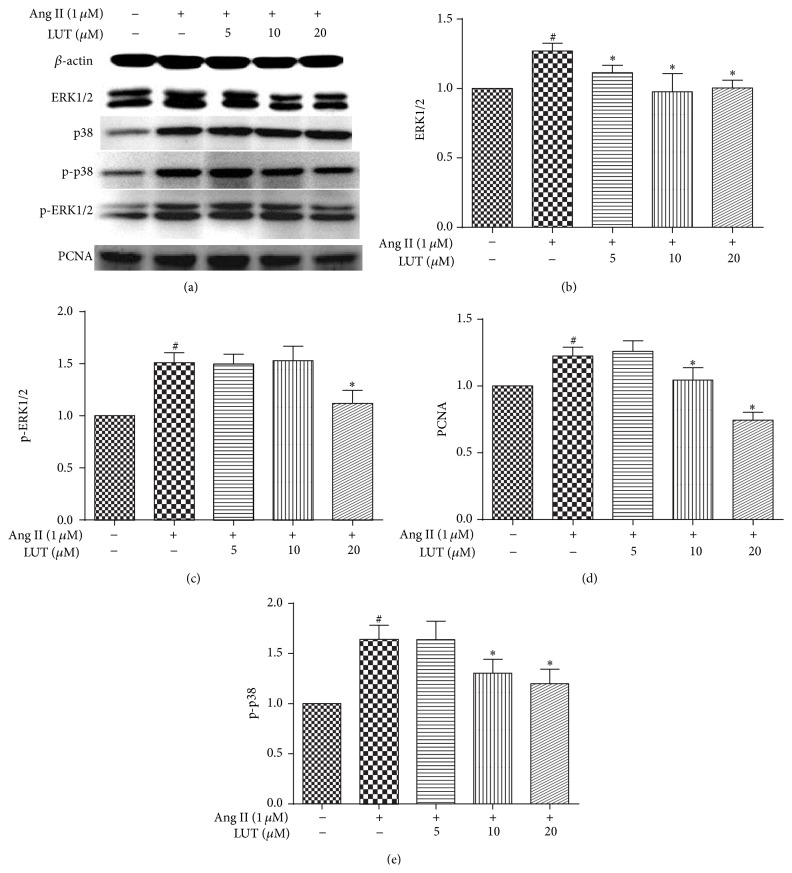
The potential drug targets for antiremodeling by luteolin. The data were expressed as mean ± SD (*n* = 3). ^#^ *P* < 0.05 versus control group; ^*∗*^ *P* < 0.05 versus Ang II-treated group.

Consequently, we examined the expression of related factors in VSMCs stimulated with Ang II for 5 min by western blot, including ERK1/2, p38, p-ERK1/2, p-p38, and PCNA. The results of western blot were presented in Figure 7. The protein expressions of ERK1/2, p38, p-ERK1/2, and p-p38 were found to be significantly higher in Ang II-treated groups compared to control. Furthermore, PCNA, a downstream factor of MAPK, was markedly upregulated. However, LUT treatment significantly inhibited the phosphorylation of ERK1/2 and p38 and decreased their activation, especially in the high concentration of LUT at 20 *μ*M. These results demonstrated that luteolin could effectively attenuate vascular remodeling through the mechanisms of downregulating the expression of Ang II, as well as suppressing the phosphorylation of ERK1/2 and p38.

## 4. Discussion

Luteolin has been shown to exhibit antihypertension activity in many experiments. Ichimura and coworkers had reported that orally administered luteolin (50 mg/kg), which is one of consistent polyphenols of the extract, significantly lowered systolic blood pressure in SHRs [18]. In some experiments, aortic rings were precontracted with phenylephrine (PE) to investigate the vasoactive effects of luteolin and its mechanisms of action on the rat thoracic aorta. They all founded that treatment with luteolin markedly inhibited the impairment of PE-induced endothelium dependent contraction in aortic rings [19–21]. Furthermore, a great number of evidences had demonstrated that luteolin suppressed the proliferation and migration of VSMCs [22]. Kim et al. tested the effects of luteolin on rat VSMCs in culture and found the antiproliferation of luteolin could act through downregulation of ERK1/2 cascade [37, 38].

As mentioned above, luteolin exhibits significant antihypertension activity as well as its inhibitory effect on the proliferation and migration of VSMCs. However, the effect of luteolin on hypertensive complication especially vascular remodeling and the molecular mechanisms are not fully understood. In the present study, we used SHR model to examine the blood pressure and the thickness of the aorta to evaluate whether luteolin could ameliorate the hypertensive vascular remodeling* in vivo*. Meanwhile, to identify these molecule's mechanisms of hypertensive vascular remodeling, we carried out the VSMCs' proliferation, migration, and oxidative stress by Ang II stimulation.

Results from the present study demonstrated that luteolin could decline the blood pressure and media thickness of vascular wall of SHR. It is pleasant that treatment with higher dosage of luteolin, the blood pressure of SHR was significantly decreased. Interestingly, the blood pressure of SHR was also markedly diminished, administration of lower dosage of luteolin. Of note, there was no significant difference among these four groups in inner diameter. However, the media thickness and media-to-lumen ratio were markedly declined by luteolin treatment. The media-to-lumen ratio was used as an index of aortic vascular remodeling [7]. Hence, luteolin can ameliorate hypertension and hypertensive vascular remodeling.

We investigated the molecular mechanisms underlying the antiremodeling activity of luteolin. The data showed that luteolin could inhibit VSMCs' proliferation and migration induced by Ang II. Meanwhile, luteolin treatment group exhibited a decreased level of ERK1/2, p-ERK1/2, p-p38, MMP-2, and PCNA protein and reduced ROS generation. These results strongly suggest that the inhibitory effect of luteolin on vascular remodeling may be at least in part mediated by inhibiting VSMCs proliferation through depressing the activation of ERK1/2, p38 and downregulating the expression of PCNA, or regulating VSMCs migration factors including ROS and MMP-2, although further investigation is required.

One of the key mechanisms involved in hypertensive vascular remodeling is the proliferation and migration of VSMCs. Ang II is a potent promoter of VSMCs proliferation and migration and has been implicated in vascular remodeling [34, 39]. As known, Ang II binds to AT_1_R at the cell surface and induces intracellular ROS generation influencing redox-sensitive signaling molecules, such as p38, ERK1/2, and MMPs [6]. On the other hand, Ang II binding to AT_1_R can directly activate all four of the major MAP kinases, including ERK1/2, p38, c-Jun NH2-terminal kinases (JNK), and ERK5 [12]. The activation of pathways leads to the excessive proliferation and migration of VSMCs, which can cause vascular remodeling [40].

In our studies, treatment with Ang II significantly increased the proliferation of VSMCs and promoted the migration of VSMCs from the upper chamber to the lower chamber. However, the effect of Ang II was suppressed by luteolin treatment. As a consequence, luteolin inhibited the Ang II-stimulated proliferation and migration of VSMCs, which was thought to be the major reason that the vascular remodeling was suppressed by luteolin administration.

It is well known that VSMCs proliferation plays major roles in vascular remodeling [41]. MAPK family is best characterized of the many growth-signaling pathways [42]. ERK1/2 is a key growth signaling kinase, which has been implicated in proliferation and migration of VSMCs [43]. Activated p38 can also affect cell proliferation, differentiation, and cytokine synthesis by upregulating the expression of transcription factors [44]. Ang II-stimulated activation of ERK1/2 and p38 is augmented [45]. These processes have been associated with enhanced vascular smooth muscle cell growth, inflammation, and fibrosis, as well as increased vascular contractility [6]. Pleasantly, we found that luteolin could significantly inhibit Ang II-induced activation of ERK1/2, p38.

PCNA is an intranuclear 36 kD polypeptide whose expression and synthesis are linked with cell proliferation [46]. PCNA expression is widely used as a marker of cell proliferation. In our study, we demonstrated that Ang II increased the expression of PCNA, which was suppressed by luteolin administration. Taken together, luteolin suppressed the activation of ERK1/2 and p38 and then declined the PCNA expression and regulated cellular proliferation in VSMCs.

Moreover, ROS plays a crucial role in Ang II-induced proliferation and migration of VSMCs [47, 48]. In the present study, we confirmed the increase in oxidative stress on cultured VSMCs stimulated by Ang II. Luteolin administration inhibited the Ang II-induced oxidative stress detected by DCFH-DA staining. As known, ROS production leads to oxidative stress, which can activate MMPs [49]. MMPs are a family of structurally related, zinc-containing enzymes that degrade the extracellular matrix and connective tissue proteins. The proteolytic effects of MMPs play an important role in cellular migration and vascular remodeling [50, 51]. We found the increase in the activity of both Pro-MMP 2 and MMP-2 in SHR. However, luteolin administration decreased Pro-MMP 2 and MMP-2 enzyme activities detected by gelatin zymography.

Another important mechanism of attenuating hypertensive vascular remodeling of luteolin is that it can suppress RAAS system, directly resulting in the decrease of Ang II expression. The renin-angiotensin system plays an important role in regulating pathophysiological processes of cardiovascular disease. Ang II, one of the most important factors in the RAAS, is a potent vasoactive peptide that causes blood vessels to constrict, resulting in increased blood pressure [28]. Meanwhile, Ang II plays a crucial role in promoting vascular remodeling [52]. In our studies, administration of luteolin, increased Ang II level in serum of SHR, was apparently declined.

**Figure 8:**
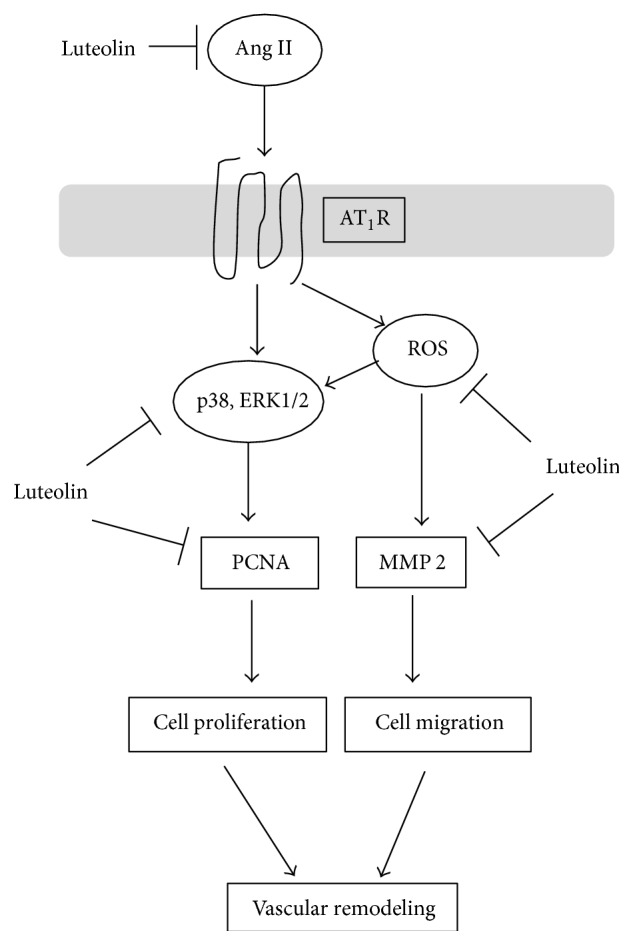
The influence of luteolin on antiremodeling related factors.

Taken together, as shown in Figure 8, our studies indicate that luteolin is a potential inhibitor of hypertensive vascular remodeling by the inhibitory effect on the proliferation and migration of VSMCs.

## 5. Conclusion

In the present study, the potential antiremodeling and antihypertensive effect of luteolin are investigated. The results suggest that luteolin is capable of directly lowering arterial blood pressure of SHR. Furthermore, luteolin can decline media thickness of vascular wall in SHR and attenuate hypertensive vascular remodeling. The antiremodeling effect of luteolin is partially associated with the suppression of VSMCs' proliferation and migration, which owes to the depression of RAAS system, directly resulting in the decrease of Ang II expression, as well as the regulation of ROS production and MAPK pathway. In short, luteolin attenuates hypertensive vascular remodeling and has the potential to be an antihypertensive drug.

## Figures and Tables

**Table 1:** The effect of luteolin on cell cycle of VSMCs stimulated by Ang II.

Distribution of cell cycle	Control	Ang II 1 *μ*M	Ang II (1 *μ*M)		
			LUT	LUT	LUT
			5 *μ*M	10 *μ*M	20 *μ*M
G_0_/G_1_	70.56%	68.09%	67.53%	69.79%	72.97%
G_2_/M	3.15%	1.87%	2.83%	2.68%	2.79%
S	25.63%	30.22%	29.82%	24.72%	24.27%
